# Association between triglyceride–glucose index and all-cause and cardiovascular mortality in US adults: A cohort study

**DOI:** 10.1097/MD.0000000000043897

**Published:** 2025-08-22

**Authors:** Chen Yan Yue, Hui Sheng Dong, Huan Xian Liu, An Mu Xie, Jing Wang

**Affiliations:** a Department of Neurology, Qingdao University Affiliated Hospital, Shandong, China; b Department of Neurology, Cao County People’s Hospital, Heze, Shandong, China; c Department of Respiratory, Cao County People’s Hospital, Heze, Shandong, China; d Department of Neurology, Chinese PLA General Hospital, Beijing, China.

**Keywords:** age, cohort study, mortality, NHANES, triglyceride–glucose index

## Abstract

The aim of this study was to investigate the association between the triglyceride–glucose (TyG) index and all-cause mortality and cardiovascular mortality, and to assess age differences. We analyzed data from the National Health and Nutrition Examination Survey (1999–2018), which included 101,316 participants. Restricted cubic spline and Cox regression models were used to examine the relationship between the TyG index and mortality rates in a sample of adult patients in the United States, age analysis was performed. Subgroup analysis was performed to evaluate potential differences in the relationship between the TyG index and mortality rates in different subgroups. The final analysis included 21,959 individuals. Observed were 3269 all-cause mortalities and 846 cardiovascular mortalities. Elevated TyG index values were associated with a significant rise in mortality, as depicted by Kaplan–Meier curves for both all-cause and cardiovascular causes (all-cause mortality: *P* < .001; cardiovascular mortality: *P* < .001). Analysis using restricted cubic splines uncovered a nonlinear association between baseline TyG index and both all-cause and cardiovascular mortality rates, with highly significant statistical correlations (all-cause mortality: *P* < .001; cardiovascular mortality: *P* = .004), with thresholds of 9.47 and 9.427. Participants were further categorized by age and divided into quartiles for survival curve analysis. Within the ≥ 40 < 60 age group, survival analyses revealed pronounced differences in mortality rates across quartiles (all-cause: *P* < .001; cardiovascular: *P* < .001). This study found a significant positive correlation between the TyG index and the overall mortality rate as well as the cardiovascular disease mortality rate among adults in the United States aged ≥ 40 and < 60.

## 1. Introduction

Amidst the backdrop of an expedited aging demographic, the incidence and mortality rates of cardiovascular disease (CVD) are on the rise, with CVD emerging as a predominant public health challenge.^[1–3]^ According to the World Health Organization, CVD is the leading cause of death from noncommunicable diseases.^[4]^ Identifying residual risk factors in individuals with CVD, particularly those with different glycemic profiles, is critical to reducing mortality, particularly by reducing the risk of cardiovascular death.

In clinical practice, insulin resistance (IR), also referred to as syndrome X or insulin resistance syndrome, denotes a condition characterized by diminished sensitivity and responsiveness to insulin’s actions, which has been associated with an increased risk of CVD.^[5,6]^ Research indicates that IR-induced hyperinsulinemia accelerates fatty acid synthesis, disrupts insulin’s normal functions, and can precipitate early atherosclerosis, atherogenic dyslipidemia, dysglycemia, and abnormal blood pressure.^[7]^ Prolonged metabolic dysregulation augments the risk of CVD mortality and, ultimately, all-cause mortality in affected patients. Consequently, the timely detection of insulin resistance is essential for initiating prompt therapeutic interventions.

Although the euglycemic hyperinsulinemic clamp and the intravenous glucose tolerance test are the gold standards for assessing IR, their invasiveness and cost prohibit routine clinical application.^[8]^ At present, the Homeostasis Model Assessment of Insulin Resistance is a prevalent method for assessing IR; however, it encounters limitations, particularly in patients undergoing insulin therapy or those with impaired β-cell function.^[9]^ A plethora of research has demonstrated that the triglyceride–glucose (TyG) index outperforms Homeostasis Model Assessment of Insulin Resistance in the assessment of IR, offering a more reliable and cost-effective alternative.^[10]^ Extensive research has demonstrated an association between the TyG index and the development and prognosis of conditions such as cardiovascular and cerebrovascular disease.^[11–14]^ However, the literature is not uniform in its findings; some studies have reported no significant correlation between the TyG index and all-cause or cardiovascular mortality,^[15]^ while others have indicated a U-shaped relationship.^[16]^ The variability of the results has led to a discussion about the practical value of the TyG index in clinical practice. Furthermore, it has been suggested that younger individuals with high TyG levels may be at increased risk of IR-related morbidity and mortality.^[17]^ On the other hand, initial research has suggested that the association between the TyG index and mortality may be more significant in older individuals.^[18]^ As such, the influence of age on the correlation between the TyG index and all-cause and cardiovascular mortality is a contentious issue that requires further elucidation. It is with this in mind that our retrospective cohort study aims to assess the predictive value of the TyG index for all-cause and cardiovascular mortality among US adults, while also scrutinizing the potential modifying effects of age on this relationship.

## 2. Methods

### 2.1. Study population and design

**Figure 1.**
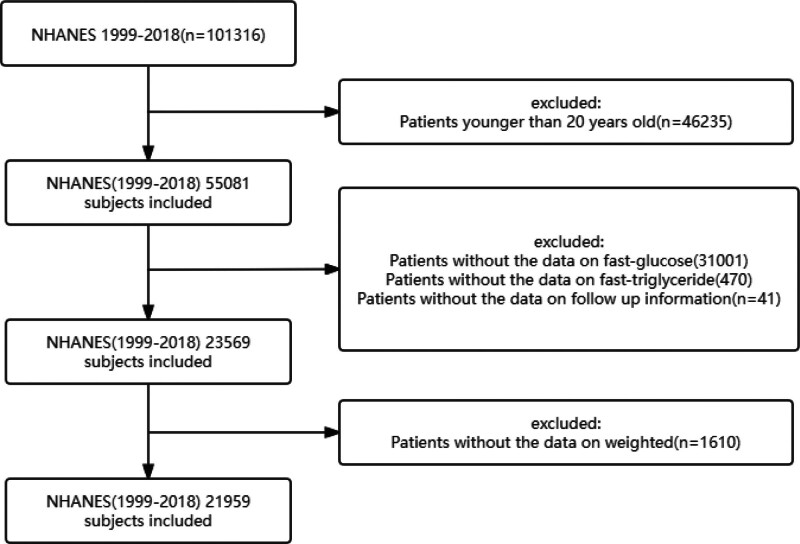
The participant enrollment procedure.

The National Health and Nutrition Examination Survey (NHANES) is a pivotal epidemiological study assessing the health and nutritional status of the U.S. population, including both adults and children. Conducted by the Centers for Disease Control and Prevention (CDC), which is tasked with generating national health statistics, NHANES adheres to protocols that have been officially sanctioned by the National Center for Health Statistics (NCHS) Research Ethics Review Board. To ensure the protection of participants’ rights, NHANES has obtained written informed consent from all individuals participating in the study. In addition, the data sets generated and analyzed in the current study are readily available on the official NHANES Website (https://www.cdc.gov/nchs/nhanes/index.html). In this cohort study, information was obtained from adult participants in the 1999 to 2018 NHANES cycles. Of the 101,316 participants, 21,959 were finally included in the analyses (Fig. 1). The following patient groups were excluded from the study: (1) patients younger than 20 years (n = 46235), (2) patients without fasting glucose data (n = 31001) and without fasting triglyceride data (n = 470), (3) patients with insufficient follow-up data (n = 41), (4) individuals with missing weight data (n = 1610).

### 2.2. Assessment of covariates

Demographic and health-related data, along with laboratory findings, were extracted from the NHANES household interviews. This comprehensive dataset encompasses variables such as age, gender, ethnicity, education, body mass index (BMI), smoking status, alcohol intake, physical activity (PA), and the presence of CVD, hypertension, hyperlipidemia, diabetes, alanine aminotransferase (ALT), aspartate aminotransferase (AST), creatinine, uric acid, triglycerides, and fasting blood glucose. Race was categorized as White, Black, Mexican, other Hispanic, or other race. Education was categorized as less than high school, high school or equivalent, or more than high school. Household income and poverty rate were categorized as ≤ 1.30, 1.31 to 3.50, >3.50. Smoking and drinking statuses were classified into 3 categories: never, former, and current. BMI was determined using the formula: weight in kilograms divided by the square of height in meters. PA (MET-min/wk) = MET × weekly frequency × duration of each PA. PA = 0 indicates participants who do not engage in any PA, otherwise, it means that participants have constant or intermittent PA. Self-reported CVD history encompassed angina pectoris, congestive heart failure, coronary heart disease, myocardial infarction, and cerebrovascular accident (stroke).

### 2.3. Assessment of TyG index

The TyG index was calculated as TyG index = Ln [fasting TG (mg/dL) × fasting glucose (mg/dL)/2]. Participants were divided into 4 groups (Q1, Q2, Q3, and Q4) according to the quartiles of the TyG index, and the Q1 group was used as the reference group.

### 2.4. Ascertainment of mortality

The study’s primary outcome was all-cause mortality, with cardiovascular mortality as the secondary outcome. To determine the mortality status of the follow-up population, we used the NHANES public-use linked mortality file as of December 31, 2019. This file was linked to the National Death Index (NDI) by the National Center for Health Statistics (NCHS) using a probability matching algorithm. In addition, we used the International Statistical Classification of Diseases, 10th Revision (ICD-10) was used to identify disease-specific deaths.

### 2.5. Statistical analysis

Due to the complex sampling design of NHANES, weighted analysis was conducted.

Study participants were divided into 4 groups according to quartiles (Q1–Q4) of the TyG index. Categorical data are presented as counts (n) and percentages (%), with chi-squared tests employed for their assessment. Continuous variables are depicted as mean ± standard deviation for normally distributed data or median (interquartile range) for skewed distributions.

We utilized Kaplan–Meier (K–M) curves and Cox proportional hazards models to examine the association between the TyG index and the risk of all-cause mortality and cardiovascular mortality. Three models were used in the multivariable Cox proportional hazards regression analysis to adjust for confounding factors. The unadjusted model is referred to as the crude model, while model 1 included age, gender and race. Model 2 was further adjusted for age, gender, race, marital status, income, education, PA, BMI, smoking, alcohol consumption, CVD, hypertension, dyslipidemia, diabetes, AST, ALT, creatinine, and uric acid. Single imputation was used for covariates with missing values and the analysis was weighted with sample weights. We use wtsaf2yr and wtsaf4yr to calculate new weight variables for the weighted analysis. Detailed information on the survey sample design and methods for calculating weights can be found at https://wwwn.cdc.gov/nchs/nhanes/analyticguidelines.aspx.

We used Cox proportional hazards regression models with restricted cubic splines and smooth curve fitting using the penalized spline method to examine the relationship between the TyG index and mortality. Two-piecewise Cox proportional hazards models were used to examine the association between the TyG index and the risk of all-cause mortality and CVD mortality on either side of the inflection point. Furthermore, to further elucidate the interaction of age with the TyG index and mortality, RCS curves and K–M curves were also analyzed in different age groups. Additionally, we also performed stratified analyses by age group (≥20 < 40 years, ≥40 < 60 years, and ≥ 60<85 years), smoking, and alcohol consumption. Outcomes are expressed as hazard ratios (HR) with 95% confidence intervals (CI). Statistical significance was set at *P* < .05 for all two-tailed tests. Analyses were conducted utilizing R statistical software (http://www.R-project.org, The R Foundation) and Free Statistics Software version 1.9.2.

## 3. Result

### 3.1. Baseline characteristics of study participants

There were 3269 (14.9%) individuals with all-cause mortality; 846 (3.9%) individuals with CVD mortality. The average age of the study participants was 49.7 ± 18.0 years. Among them, 51.9% were female, and 48.1% were male. Baseline patient characteristics were stratified according to quartiles (Q) of the TyG index: Q1: 5.647 to 8.196; Q2: 8.196 to 8.609; Q3: 8.609 to 9.054; Q4: 9.054 to 13.405, respectively. Patients with higher TyG index values were more likely to be White Hispanic, married, current alcohol drinkers, and dyslipidemic compared to those with lower TyG index values (Table S1, Supplemental Digital Content, https://links.lww.com/MD/P680).

### 3.2. K–M survival analysis curves for all-cause and cardiovascular mortality according to TyG index

**Figure 2.**
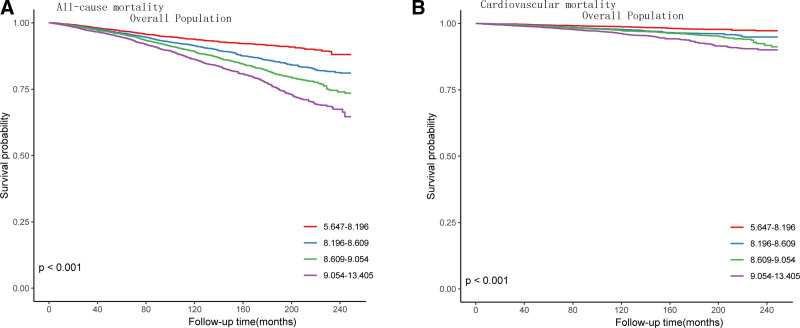
Kaplan–Meier survival analysis curves for all-cause and cardiovascular mortality. TyG index quartile: 5.647 to 8.196; 8.196 to 8.609; 8.609 to 9.054; 9.054 to 13.405. Kaplan–Meier analysis for mortality among TyG index groups in (A) all-cause mortality, (B) cardiovascular mortality.

During the 13-year follow-up period, there were 3269 cases of all-cause mortality and 846 cases of cardiovascular mortality; there were significant differences in mortality rates among different TyG index groups (all-cause mortality: *P* < .001, Fig. 2A; cardiovascular mortality: *P* < .001, Fig. 2B) in the total population.

### 3.3. Associations of triglyceride glucose index with all-cause and cardiovascular mortality

We constructed 3 Cox regression models to examine the independent association between TyG index levels and the risk of mortality in (Table 1). The crude model was unadjusted, model 1 adjusted for age, gender, race; model 2 adjusted for age, gender, race, marital status, income, education, PA, BMI, smoking, alcohol consumption, CVD, hypertension, dyslipidemia, diabetes, AST, ALT, creatinine, and uric acid. In the unadjusted model, TyG was significantly and positively associated with both all-cause mortality and CVD deaths (*P* < .001), and similar results were seen in model 1 when TyG was used as a continuous variable (all-cause mortality: HR (95% CI) 1.26 (1.17–1.35); cardiovascular mortality: HR (95% CI) 1.43 (1.23, 1.66)). When TyG was divided into quartiles, the positive association with all-cause mortality and CVD mortality was more pronounced in groups with higher TyG (all-cause mortality: HR (95% CI) 1.22 (1.06–1.40); cardiovascular mortality: HR (95% CI) 1.52 (1.13, 2.03)).

**Table 1:** HR (95% CI) for outcomes across groups of the triglyceride–glucose index.

	Crude HR (95% CI)	*P*-value	Model 1 HR (95% CI)	*P*-value	Model 2 HR (95% CI)	*P*-value
All-cause mortality						
Continuous	1.62 (1.52, 1.72)	<.001	1.26 (1.17, 1.35)	<.001	1.07 (0.99, 1.15)	.096
Quartiles						
Q1	1		1		1	
Q2	1.55 (1.34, 1.80)	<.001	0.92 (0.79, 1.05)	.222	0.92 (0.79, 1.06)	.231
Q3	2.03 (1.75, 2.35)	<.001	0.99 (0.85, 1.14)	.84	0.86 (0.74, 1.00)	.05
Q4	2.67 (2.30, 3.11)	<.001	1.22 (1.06, 1.40)	.005	0.95 (0.82, 1.10)	.474
Cardiovascular mortality						
Continuous	1.76 (1.58, 1.97)	<.001	1.43 (1.23, 1.66)	<.001	1.05 (0.88, 1.25)	.585
Quartiles						
Q1	1		1		1	
Q2	1.83 (1.35, 2.47)	<.001	1.03 (0.77, 1.39)	.822	1.00 (0.75, 1.34)	.997
Q3	2.24 (1.70, 2.96)	<.001	1.06 (0.81, 1.40)	.662	0.79 (0.58, 1.07)	.133
Q4	3.39 (2.52, 4.54)	<.001	1.52 (1.13, 2.03)	.005	0.91 (0.67, 1.25)	.567

HR (95% CI) for outcomes across groups of the TyG index; model 1 adjusted for age, sex, race; model 2 adjusted for age, gender, race, marital status, income, education, physical activity, BMI, smoking, alcohol consumption, CVD, hypertension, dyslipidemia, diabetes, AST, ALT, creatinine, and uric acid. Baseline patient characteristics were stratified according to quartiles (Q) of the TyG index: Q1: 5.647 to 8.196; Q2: 8.196 to 8.609; Q3: 8.609 to 9.054; Q4: 9.054 to 13.405.

ALT = alanine aminotransferase, AST = aspartate aminotransferase, BMI = body mass index, CI = confidence interval, CVD = cardiovascular disease, DM = diabetes mellitus, HR = hazard ratio, TyG index = triglyceride–glucose index.

### 3.4. The detection of nonlinear relationships

**Figure 3.**
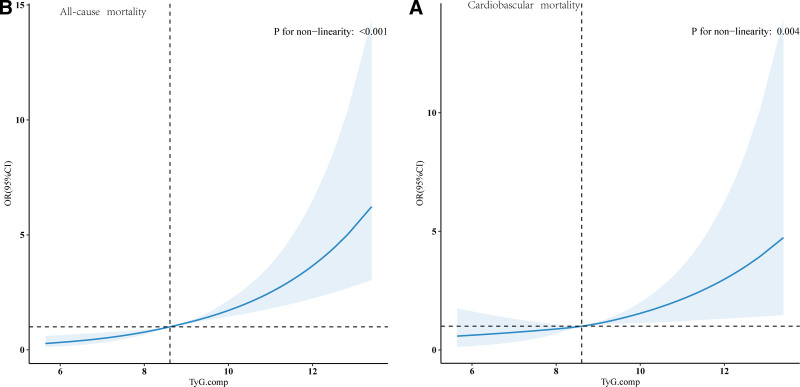
The association between the TyG index and all-cause mortality (A) and cardiovascular mortality (B) in the general population was assessed. Adjustments were made for age, gender, race, marital status, income, education, physical activity, BMI, smoking, alcohol consumption, CVD, hypertension, dyslipidemia, diabetes, AST, ALT, creatinine, and uric acid. The solid line and the blue area represent the estimated values and their corresponding 95% CIs. ALT = alanine aminotransferase, AST = aspartate aminotransferase, BMI = body mass index, CVD = cardiovascular disease, DM = diabetes mellitus, TyG index = triglyceride–glucose index.

Given the multivariate Cox proportional hazards analysis indicated a nonlinear relationship between the baseline TyG index and both all-cause and cardiovascular mortality, we employed restricted cubic spline analysis to delve deeper into this relationship. The adjusted spline plots revealed significant nonlinear associations between the TyG index and all-cause mortality (*P* < .001, Fig. 3A) as well as cardiovascular mortality (*P* = .004, Fig. 3B).

Based on a two-stage Cox proportional risk regression model, we found inflection points of 9.47 and 9.427 for all-cause mortality and CVD mortality, respectively (*P*-values of both log-likelihood ratios were <.05, Table 2). After adjustment for age, sex, race, marital status, income, education, PA, BMI, smoking, alcohol consumption, CVD, hypertension, dyslipidemia, diabetes mellitus (DM), ghrelin, creatinine, and uric acid, the all-cause mortality rate tended to increase with TyG > 9.47, with a 40.6% increase in all-cause mortality for each increase in TyG; and the all-cause mortality tended to increase with TyG > 9.427. For TyG > 9.427, the risk of CVD increased by 79.5% for each unit increase in TyG.

**Table 2:** Threshold effect analysis of triglyceride-glucose index on all-cause and CVD mortality.

All-cause mortality		
TyG index	HR (95% CI)	*P*-value
Inflection point	9.47 (9.372, 9.568)	NA
TyG index < 9.47	0.934 (0.85, 1.028)	.1616
TyG index ≥ 9.47	1.406 (1.08, 1.83)	.0114
*P* for log-likelihood ratio	–	<.001
CVD mortality		
TyG index	HR (95% CI)	*P*-value
Inflection point	9.427 (9.394, 9.461)	NA
TyG index < 9.427	0.896 (0.74, 1.086)	.2631
TyG index ≥ 9.427	1.795 (1.136, 2.836)	.0122
*P* for log-likelihood ratio	–	<.001

Cox proportional hazards models were used to estimate HR and 95% CI. Adjusted for age, sex, race, marital status, income, education, physical activity, BMI, smoking, alcohol consumption, CVD, hypertension, dyslipidemia, diabetes, AST, ALT, creatinine, and uric acid.

ALT = alanine aminotransferase, AST = aspartate aminotransferase, BMI = body mass index, CI = confidence interval, CVD = cardiovascular disease, DM = diabetes mellitus, HR = hazard ratio, TyG index = triglyceride–glucose index.

### 3.5. Subgroup analysis of the association between TyG index and all-cause and cardiovascular mortality

Table 3 shows the results of the subgroup analysis. Stratified analysis by age, gender, and race showed an interaction within the age subgroups. The study results showed a strong association between the TyG index and all-cause and cardiovascular mortality in the ≥ 40 < 60 age group (all-cause mortality: HR (95% CI) 1.29 (1.06, 1.58), *P* = .01; cardiovascular mortality: HR (95% CI) 1.45 (1.04, 2.04), *P* = .03). However, there was no association in the age group ≥ 20 < 40 years (all-cause mortality: HR (95% CI) 1.23 (0.85, 1.80), *P* = .27; cardiovascular mortality: HR (95% CI) 1.12 (0.09, 1. 12), *P* = .13) or the ≥ 60 < 85 age group (all-cause mortality: HR (95% CI) 0.98 (0.90, 1.06), *P* = .64; cardiovascular mortality: HR (95% CI) 0.85 (0.70, 1.03), *P* = .09). No significant interaction was observed in the smoking and drinking subgroups.

**Table 3:** Subgroup analysis of the association between the triglyceride-glucose index and all-cause mortality and cardiovascular mortality.

Variables	HR (95% CI)	*P*-value	*P* for interaction
All-cause mortality			
Age			<.001
≥20<40	1.23 (0.85, 1.80)	.27	
≥40<60	1.29 (1.06, 1.58)	.01	
≥60	0.98 (0.90, 1.06)	.64	
Gender			.867
Male	1.08 (0.97, 1.20)	.15	
Female	1.05 (0.94, 1.18)	.38	
Smoke			.105
Never	1.22 (1.05, 1.40)	.01	
Former	0.98 (0.85, 1.13)	.77	
Now	1.03 (0.87, 1.22)	.72	
Drink			.651
Never	1.14 (0.90, 1.44)	.27	
Former	1.00 (0.87, 1.15)	.99	
Now	1.02 (0.90, 1.15)	.76	
Cardiovascular mortality			
Age			<.001
≥20<40	1.12 (0.09, 1.12)	.13	
≥40<60	1.45 (1.04, 2.04)	.03	
>60	0.85 (0.70, 1.03)	.09	
Gender			.24
Male	1.13 (0.93, 1.37)	.21	
Female	0.92 (0.69, 1.21)	.54	
Smoke			.628
Never	1.08 (0.80, 1.45)	.61	
Former	1.07 (0.78, 1.46)	.68	
Now	0.95 (0.68, 1.32)	.75	
Drink			.628
Never	1.01 (0.68, 1.52)	.95	
Former	1.11 (0.82, 1.50)	.51	
Now	0.97 (0.78, 1.21)	.82	

Subgroup analysis of the association between TyG index and CVD mortality. Adjusted for race, marital status, income, education, physical activity, BMI, CVD, hypertension, hyperlipidemia, diabetes, AST, ALT, creatinine, and uric acid, excluding the subgroup factor itself.

ALT = alanine aminotransferase, AST = aspartate aminotransferase, BMI = body mass index, CI = confidence interval, CVD = cardiovascular disease, DM = diabetes mellitus, HR = hazard ratio, TyG index = triglyceride–glucose index.

### 3.6. K–M survival analysis curves for all-cause and cardiovascular mortality stratified by age and based on the TyG index

Further stratified analysis by age (≥20 < 40, ≥40 < 60, ≥60 < 85) showed significant differences in mortality rates in the ≥ 40 < 60 age group (all-cause mortality: *P* < .001; cardiovascular mortality: *P* < .001, Figure S1A and S1B, Supplemental Digital Content, https://links.lww.com/MD/P679). However, the differences in mortality rates in the ≥ 20 < 40 age group did not reach statistical significance (all-cause mortality; *P* = .139; cardiovascular mortality: *P* = .294, Figure S1C and S1D, Supplemental Digital Content, https://links.lww.com/MD/P679). Similarly, in the ≥ 60 < 85 age group, the differences in mortality rates did not reach statistical significance (all-cause mortality; *P* = .905; cardiovascular mortality: *P* = .499, Figure S1E and S1F, Supplemental Digital Content, https://links.lww.com/MD/P679).

## 4. Discussion

This study demonstrated a nonlinear relationship between the TyG index and all-cause and cardiovascular mortality in the general population. Our study suggests that the TyG index is a reliable predictor of all-cause mortality and cardiovascular mortality. In addition, a notable interaction between the TyG index and age was observed, indicating that the association between TyG levels and all-cause and cardiovascular mortality was more pronounced in individuals aged ≥ 40 and < 60 years.

Our study’s observation of a nonlinear relationship between the TyG index and all-cause and CVD mortality aligns partially with recent research by Liu, Qin, and Zhang et al.^[19–22]^ Liu and colleagues identified a nonlinear correlation between the TyG index and mortality, encompassing both all-cause and CVD mortality,^[19]^ while Qin and Zhang team noted a U-shaped link between the baseline TyG index and all-cause mortality among CVD patients in the U.S. population.^[20]^ Han Zhang and colleagues described a nonlinear association between the TyG index and mortality attributable to CVD and other factors in patients with diabetic kidney disease^[21]^; importantly, they did not stratify their analysis by age. Conversely, Zhou D and colleagues reported a nonlinear association between all-cause mortality and the TyG index in elderly hypertensive patients aged 60 years and older.^[22]^ Jiaqi Chen et al discovered significant correlations between the TyG index and all-cause and cardiovascular mortality, particularly in individuals below the age of 65.^[8]^ Although both studies considered age subgroups, the outcomes were not entirely consistent.

In our current investigation, we discerned age-dependent variations in the correlation between the TyG index and mortality, a finding that aligns with prior research. Younan Yao et al observed that within a national cohort of patients with type 2 diabetes mellitus (T2DM) in the United States, the relationship between the TyG index and all-cause/non-cardiovascular (CV) mortality was age-dependent, with elevated TyG values being linked to an increased risk of all-cause/non-CV mortality specifically in T2DM patients under the age of 65.^[23]^ This increased risk was not seen in older patients. Similarly, the TyG index has been identified as an independent predictor of both all-cause and cause-specific mortality in the general population, highlighting the importance of caution in younger individuals.^[24]^ Yun Kyung Cho et al identified the TyG index as a straightforward marker of IR that forecasts CVD and mortality in young and ostensibly healthy individuals.^[25]^ In contrast, Min Sun et al discovered a U-shaped association between the TyG index and mortality, with lower mortality rates observed in middle-aged and older individuals.^[26]^ Jian Shen reported that the TyG index predicts the risk of all-cause mortality in the oldest patients with acute coronary syndrome complicated by DM.^[27]^ After further stratification by age, our study revealed unique age-related trends in the association between the TyG index and CVD mortality. Although the exact biological mechanisms linking the TyG index to mortality are not fully understood, it is conceivable that fundamental pathways involve IR, a state characterized by reduced sensitivity to insulin. IR is recognized for its role in catalyzing the production of glycosylation products and free radicals, which in turn can decrease the bioavailability of nitric oxide. This reduction can adversely affect the vascular endothelium, compromise endothelium-mediated vasodilation, and increase the risk of various diseases.^[28]^ Firstly, chronic hyperglycemia and dyslipidemia, hallmarks of IR, can trigger oxidative stress, enhance inflammatory responses, promote foam cell formation, and disrupt endothelial function, thereby stimulating smooth muscle cell proliferation.^[29]^ Moreover, IR activates the mitochondrial electron transport chain, leading to heightened reactive oxidative species and endothelial injury. Additionally, IR disrupts metabolic homeostasis in diabetic individuals, exacerbating hyperglycemia, inflammation, oxidative stress, and lipid metabolism imbalances.^[30]^

In aggregate, our findings advocate for the TyG index as a dependable and precise measure of IR for real-world risk stratification, underscoring its potential utility in identifying individuals at increased risk of adverse health outcomes.

### 4.1. Strengths and limitations

This study is advanced because it demonstrates a potential interaction between age and the TyG index and builds on previous studies predicting all-cause and cardiovascular mortality in US adults. Moreover, our research underscores the robust correlation between the TyG index and mortality outcomes in individuals aged 40 to 60 years, offering a theoretical foundation for the utilization of the TyG index in middle-aged populations. Additionally, the study capitalizes on the strengths of a large national database, boasting a substantial sample size and an extended follow-up period.

Despite these merits, our study is not without limitations. Firstly, the post hoc analysis nature of our work means that complete elimination of bias was not feasible. Secondly, as a single-center observational study, we are constrained from establishing causal relationships. Thirdly, despite our efforts to control for confounding variables through multivariate adjustment and subgroup analyses, there is still a possibility of unaddressed residual confounding that may affect prognosis.

## 5. Conclusion

This study found a significant positive association between the TyG index and all-cause mortality and CVD mortality in the adult population in the United States, particularly in the age group ≥ 40 < 60 years.

## Acknowledgments

We gratefully thank Dr Xiao Lian Peng (Department of Obstetrics and Gynecology, People’s Hospital of Xie gang, Dongguan City, Guangdong Province) for her comments on the manuscript.

## Author contributions

**Conceptualization:** Jing Wang, An Mu Xie.

**Data curation:** Chen Yan Yue.

**Investigation:** Hui Sheng Dong.

**Methodology:** Jing Wang, An Mu Xie.

**Software:** Jing Wang, Huan Xian Liu, An Mu Xie.

**Supervision:** Jing Wang.

**Validation:** Huan Xian Liu.

**Visualization:** Hui Sheng Dong.

**Writing – original draft:** Chen Yan Yue.

**Writing – review & editing:** Chen Yan Yue, Jing Wang.

## Supplementary Material



**Figure:**
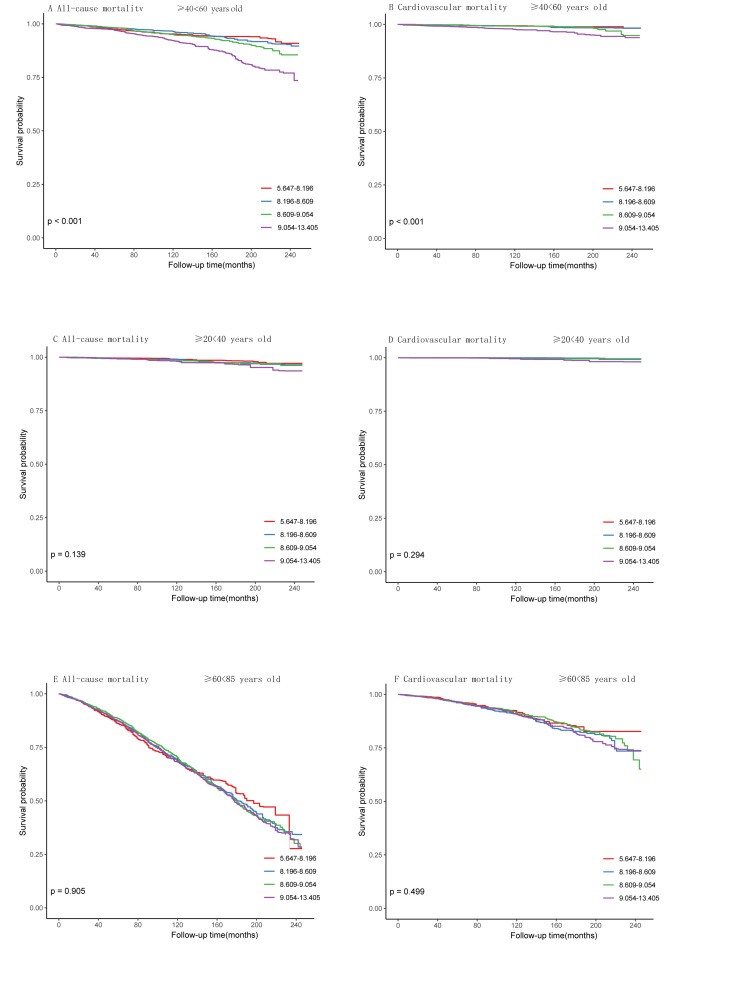


## References

[1] Virani SS Alonso A Benjamin EJ. Heart disease and stroke statistics-2020 update: a report from the American Heart Association. Circulation. 2020;141:e139–596. 31992061 10.1161/CIR.0000000000000757

[2] Vaduganathan M Mensah GA Turco JV Fuster V Roth GA. The global burden of cardiovascular diseases and risk: a compass for future health. J Am Coll Cardiol. 2022;80:2361–71. 36368511 10.1016/j.jacc.2022.11.005

[3] Liu C Liang D Xiao K Xie L. Association between the triglyceride–glucose index and all-cause and CVD mortality in the young population with diabetes. Cardiovasc Diabetol. 2024;23:171. 38755682 10.1186/s12933-024-02269-0 PMC11097545

[4] Balakumar P Maung-U K Jagadeesh G. Prevalence and prevention of cardiovascular disease and diabetes mellitus. Pharmacol Res. 2016;113:600–9. 27697647 10.1016/j.phrs.2016.09.040

[5] Tahapary DL Pratisthita LB Fitri NA. Challenges in the diagnosis of insulin resistance: focusing on the role of HOMA-IR and Tryglyceride/glucose index. Diabetes Metab Syndr. 2022;16:102581. 35939943 10.1016/j.dsx.2022.102581

[6] Mancusi C de Simone G Best LG. Myocardial mechano-energetic efficiency and insulin resistance in non-diabetic members of the Strong Heart Study cohort. Cardiovasc Diabetol. 2019;18:56. 31039789 10.1186/s12933-019-0862-9 PMC6492323

[7] Badmus OO Hillhouse SA Anderson CD Hinds TD Stec DE. Molecular mechanisms of metabolic associated fatty liver disease (MAFLD): functional analysis of lipid metabolism pathways. Clin Sci (Lond). 2022;136:1347–66. 36148775 10.1042/CS20220572 PMC9508552

[8] Chen J Wu K Lin Y Huang M Xie S. Association of triglyceride glucose index with all-cause and cardiovascular mortality in the general population. Cardiovasc Diabetol. 2023;22:320. 37993902 10.1186/s12933-023-02054-5 PMC10666367

[9] Tao LC Xu JN Wang TT Hua F Li JJ. Triglyceride-glucose index as a marker in cardiovascular diseases: landscape and limitations. Cardiovasc Diabetol. 2022;21:68. 35524263 10.1186/s12933-022-01511-x PMC9078015

[10] Park HM Lee HS Lee YJ Lee JH. The triglyceride–glucose index is a more powerful surrogate marker for predicting the prevalence and incidence of type 2 diabetes mellitus than the homeostatic model assessment of insulin resistance. Diabetes Res Clin Pract. 2021;180:109042. 34506839 10.1016/j.diabres.2021.109042

[11] Hoshino T Mizuno T Ishizuka K. Triglyceride–glucose index as a prognostic marker after ischemic stroke or transient ischemic attack: a prospective observational study. Cardiovasc Diabetol. 2022;21:264. 36451149 10.1186/s12933-022-01695-2 PMC9714168

[12] Ding X Wang X Wu J Zhang M Cui M. Triglyceride–glucose index and the incidence of atherosclerotic cardiovascular diseases: a meta-analysis of cohort studies. Cardiovasc Diabetol. 2021;20:76. 33812373 10.1186/s12933-021-01268-9 PMC8019501

[13] Guo X Shen R Yan S Su Y Ma L. Triglyceride–glucose index for predicting repeat revascularization and in-stent restenosis in patients with chronic coronary syndrome undergoing percutaneous coronary intervention. Cardiovasc Diabetol. 2023;22:43. 36864455 10.1186/s12933-023-01779-7 PMC9983161

[14] Cai W Xu J Wu X. Association between triglyceride–glucose index and all-cause mortality in critically ill patients with ischemic stroke: analysis of the MIMIC-IV database. Cardiovasc Diabetol. 2023;22:138. 37312120 10.1186/s12933-023-01864-x PMC10262584

[15] Liu X Tan Z Huang Y. Relationship between the triglyceride–glucose index and risk of cardiovascular diseases and mortality in the general population: a systematic review and meta-analysis. Cardiovasc Diabetol. 2022;21:124. 35778731 10.1186/s12933-022-01546-0 PMC9250255

[16] Yu Y Wang J Ding L. Sex differences in the nonlinear association of triglyceride glucose index with all-cause and cardiovascular mortality in the general population. Diabetol Metab Syndr. 2023;15:136. 37349808 10.1186/s13098-023-01117-7 PMC10288670

[17] Liu R Li L Wang L Zhang S. Triglyceride–glucose index predicts death in patients with stroke younger than 65. Front Neurol. 2023;14:1198487. 37602260 10.3389/fneur.2023.1198487 PMC10435085

[18] Pang J Qian L Che X Lv P Xu Q. TyG index is a predictor of all-cause mortality during the long-term follow-up in middle-aged and elderly with hypertension. Clin Exp Hypertens. 2023;45:2272581. 37902269 10.1080/10641963.2023.2272581

[19] Liu XC He GD Lo K Huang YQ Feng YQ. The triglyceride–glucose index, an insulin resistance marker, was non-linear associated with All-cause and cardiovascular mortality in the general population. Front Cardiovasc Med. 2021;7:628109. 33521071 10.3389/fcvm.2020.628109 PMC7840600

[20] Zhang Q Xiao S Jiao X Shen Y. The triglyceride–glucose index is a predictor for cardiovascular and all-cause mortality in CVD patients with diabetes or pre-diabetes: evidence from NHANES 2001–2018. Cardiovasc Diabetol. 2023;22:279. 37848879 10.1186/s12933-023-02030-z PMC10583314

[21] Zhang H Wang L Zhang Q. Non-linear association of triglyceride–glucose index with cardiovascular and all-cause mortality in T2DM patients with diabetic kidney disease: NHANES 2001–2018 retrospective cohort study. Lipids Health Dis. 2024;23:253. 39154178 10.1186/s12944-024-02249-z PMC11330591

[22] Zhou D Liu XC Kenneth L Huang YQ Feng YQ. A Non-linear association of triglyceride glycemic index with cardiovascular and all-cause mortality among patients with hypertension. Front Cardiovasc Med. 2022;8:778038. 35155598 10.3389/fcvm.2021.778038 PMC8828937

[23] Yao Y Wang B Geng T Chen J Chen W Li L. The association between TyG and all-cause/non-cardiovascular mortality in general patients with type 2 diabetes mellitus is modified by age: results from the cohort study of NHANES 1999–2018. Cardiovasc Diabetol. 2024;23:43. 38281973 10.1186/s12933-024-02120-6 PMC10823741

[24] Li S An L Fu Z Zhang W Liu H. Association between triglyceride–glucose related indices and all-cause and cause-specific mortality in the general population: a cohort study. Cardiovasc Diabetol. 2024;23:286. 39113049 10.1186/s12933-024-02390-0 PMC11304911

[25] Cho YK Han KD Kim HS Jung CH Park JY Lee WJ. Triglyceride–glucose index is a useful marker for predicting future cardiovascular disease and mortality in young Korean adults: a nationwide population-based cohort study. J Lipid Atheroscler. 2022;11:178–86. 35656153 10.12997/jla.2022.11.2.178 PMC9133778

[26] Sun M Guo H Wang Y Ma D. Association of triglyceride glucose index with all-cause and cause-specific mortality among middle age and elderly US population. BMC Geriatr. 2022;22:461. 35643423 10.1186/s12877-022-03155-8 PMC9145102

[27] Shen J Feng B Fan L. Triglyceride glucose index predicts all-cause mortality in oldest-old patients with acute coronary syndrome and diabetes mellitus. BMC Geriatr. 2023;23:78. 36747129 10.1186/s12877-023-03788-3 PMC9901061

[28] Molina MN Ferder L Manucha W. Emerging role of nitric oxide and heat shock proteins in insulin resistance. Curr Hypertens Rep. 2016;18:1. 26694820 10.1007/s11906-015-0615-4

[29] Gao S Ma W Huang S Lin X Yu M. Impact of triglyceride–glucose index on long-term cardiovascular outcomes in patients with myocardial infarction with nonobstructive coronary arteries. Nutr Metab Cardiovasc Dis. 2021;31:3184–92. 34511291 10.1016/j.numecd.2021.07.027

[30] Samuel VT Shulman GI. The pathogenesis of insulin resistance: integrating signaling pathways and substrate flux. J Clin Invest. 2016;126:12–22. 26727229 10.1172/JCI77812 PMC4701542

