# Supplementary material for: Association between triglyceride–glucose index and all-cause and cardiovascular mortality in US adults: A cohort study
Source: Medicine (Baltimore). 2025 Aug 22;104(34):e43897. doi: 10.1097/MD.0000000000043897 (PMC12384810; doi:10.1097/MD.0000000000043897)
Supplement: Supplementary file 1 [file medi-104-e43897-s001.doc]

Supplementary table.Baseline characteristics according to the Triglyceride-Glucose index quartiles

| **Variables** |  | **Quartiles of TyG index** | | | |  |
| --- | --- | --- | --- | --- | --- | --- |
|  | **Total** | **Q1** | **Q2** | **Q3** | **Q4** | ***p_value*** |
| **N(%)** | 21959 | 5551 | 5563 | 5501 | 5344 |  |
| **Age,years** | 49.7 ± 18.0 | 43.4 ± 17.6 | 49.5 ± 18.4 | 52.3 ± 17.8 | 53.8 ± 16.5 | < 0.001 |
| **Gender, n (%)** |  |  |  |  |  | < 0.001 |
| Male | 10556 (48.1) | 2196 (39.6) | 2649 (47.6) | 2787 (50.7) | 2924 (54.7) |  |
| Female | 11403 (51.9) | 3355 (60.4) | 2914 (52.4) | 2714 (49.3) | 2420 (45.3) |  |
| **Race, n (%)** |  |  |  |  |  | < 0.001 |
| White | 9855 (44.9) | 2208 (39.8) | 2474 (44.5) | 2605 (47.4) | 2568 (48.1) |  |
| Hispanic Black | 4239 (19.3) | 1703 (30.7) | 1198 (21.5) | 772 (14) | 566 (10.6) |  |
| Mexican American | 3966 (18.1) | 665 (12) | 924 (16.6) | 1091 (19.8) | 1286 (24.1) |  |
| Other Hispanic | 1870 ( 8.5) | 400 (7.2) | 466 (8.4) | 523 (9.5) | 481 (9) |  |
| Other Race | 2029 ( 9.2) | 575 (10.4) | 501 (9) | 510 (9.3) | 443 (8.3) |  |
| **Marry, n (%)** |  |  |  |  |  | < 0.001 |
| Married | 13562 (61.8) | 3147 (56.7) | 3379 (60.7) | 3564 (64.8) | 3472 (65) |  |
| Never married | 8397 (38.2) | 2404 (43.3) | 2184 (39.3) | 1937 (35.2) | 1872 (35) |  |
| **PIR, n (%)** |  |  |  |  |  | < 0.001 |
| ≤1.30 | 6747 (30.7) | 1595 (28.7) | 1659 (29.8) | 1701 (30.9) | 1792 (33.5) |  |
| 1.31-3.50 | 8497 (38.7) | 2127 (38.3) | 2116 (38) | 2136 (38.8) | 2118 (39.6) |  |
| >3.50 | 6715 (30.6) | 1829 (32.9) | 1788 (32.1) | 1664 (30.2) | 1434 (26.8) |  |
| **Education, n (%)** |  |  |  |  |  | < 0.001 |
| Less than high school | 5890 (26.8) | 1095 (19.7) | 1409 (25.3) | 1636 (29.7) | 1750 (32.7) |  |
| High school or equivalent | 5025 (22.9) | 1170 (21.1) | 1295 (23.3) | 1257 (22.9) | 1303 (24.4) |  |
| Above high school | 11044 (50.3) | 3286 (59.2) | 2859 (51.4) | 2608 (47.4) | 2291 (42.9) |  |
| **Smoke, n (%)** |  |  |  |  |  | < 0.001 |
| Never | 11963 (54.5) | 3493 (62.9) | 3051 (54.8) | 2890 (52.5) | 2529 (47.3) |  |
| Former | 5573 (25.4) | 1095 (19.7) | 1345 (24.2) | 1482 (26.9) | 1651 (30.9) |  |
| Now | 4423 (20.1) | 963 (17.3) | 1167 (21) | 1129 (20.5) | 1164 (21.8) |  |
| **Drink, n (%)** |  |  |  |  |  | < 0.001 |
| Never | 3163 (14.4) | 801 (14.4) | 772 (13.9) | 778 (14.1) | 812 (15.2) |  |
| Former | 4239 (19.3) | 821 (14.8) | 1000 (18) | 1154 (21) | 1264 (23.7) |  |
| Now | 14557 (66.3) | 3929 (70.8) | 3791 (68.1) | 3569 (64.9) | 3268 (61.2) |  |
| **PA** | 543.9 (0.0, 2400.0) | 840.0 (55.1, 3120.0) | 560.0 (0.0, 2457.0) | 480.0 (0.0, 2000.0) | 378.0 (0.0, 1795.5) | < 0.001 |
| **BMI,kg/m2** | 29.0 ± 6.8 | 26.6 ± 6.5 | 28.4 ± 6.7 | 29.8 ± 6.7 | 31.2 ± 6.4 | < 0.001 |
| **CVD, n (%)** |  |  |  |  |  | < 0.001 |
| No | 19542 (89.0) | 5177 (93.3) | 5029 (90.4) | 4840 (88) | 4496 (84.1) |  |
| Yes | 2417 (11.0) | 374 (6.7) | 534 (9.6) | 661 (12) | 848 (15.9) |  |
| **Hyperlipidemia, n (%)** |  |  |  |  |  | < 0.001 |
| No | 5812 (26.5) | 3100 (55.8) | 1788 (32.1) | 831 (15.1) | 93 (1.7) |  |
| Yes | 16147 (73.5) | 2451 (44.2) | 3775 (67.9) | 4670 (84.9) | 5251 (98.3) |  |
| **Hypertension, n (%)** |  |  |  |  |  | < 0.001 |
| No | 12752 (58.1) | 4005 (72.1) | 3399 (61.1) | 2930 (53.3) | 2418 (45.2) |  |
| Yes | 9207 (41.9) | 1546 (27.9) | 2164 (38.9) | 2571 (46.7) | 2926 (54.8) |  |
| **DM, n (%)** |  |  |  |  |  | < 0.001 |
| No | 17854 (81.3) | 5232 (94.3) | 4969 (89.3) | 4496 (81.7) | 3157 (59.1) |  |
| Yes | 4105 (18.7) | 319 (5.7) | 594 (10.7) | 1005 (18.3) | 2187 (40.9) |  |
| **all_cause_mortality, n (%)** |  |  |  |  |  | < 0.001 |
| No | 18690 (85.1) | 5092 (91.7) | 4767 (85.7) | 4576 (83.2) | 4255 (79.6) |  |
| Yes | 3269 (14.9) | 459 (8.3) | 796 (14.3) | 925 (16.8) | 1089 (20.4) |  |
| **CVD_mortality, n (%)** |  |  |  |  |  | < 0.001 |
| No | 21113 (96.1) | 5447 (98.1) | 5355 (96.3) | 5267 (95.7) | 5044 (94.4) |  |
| Yes | 846 ( 3.9) | 104 (1.9) | 208 (3.7) | 234 (4.3) | 300 (5.6) |  |
| **Alt,U/L** | 21.0 (16.0, 28.0) | 18.0 (14.0, 23.0) | 20.0 (15.0, 26.0) | 21.0 (17.0, 29.0) | 24.0 (18.0, 34.0) | < 0.001 |
| **Ast,U/L** | 22.0 (19.0, 27.0) | 21.0 (18.0, 26.0) | 22.0 (19.0, 26.0) | 23.0 (19.0, 27.0) | 23.0 (19.0, 29.0) | < 0.001 |
| **Creatinine,mg/dl** | 0.8 (0.7, 1.0) | 0.8 (0.7, 0.9) | 0.8 (0.7, 1.0) | 0.8 (0.7, 1.0) | 0.8 (0.7, 1.0) | < 0.001 |
| **Uric_acid,mg/dl** | 5.5 ± 1.5 | 4.9 ± 1.3 | 5.4 ± 1.4 | 5.7 ± 1.4 | 5.9 ± 1.5 | < 0.001 |

Notes:Data presented are values are numbers (percentages),mean ± SD,median and IQ range;TyG index quartile (Q):Q1: 5.647-8.196; Q2: 8.196-8.609; Q3: 8.609-9.054; Q4: 9.054-13.405; Abbreviations:TyG, index triglyceride glucose index;PIR, poverty income ratio; BMI, body mass index; DM, diabetes; PA, physical activity;ALT alanine transaminase, AST aspartate transaminase; CVD cardiovascular disease.
